# Four reversible and reconfigurable structures for three-phase emulsions: extended morphologies and applications

**DOI:** 10.1038/srep42738

**Published:** 2017-02-15

**Authors:** Xue-hui Ge, Yu-hao Geng, Qiao-chu Zhang, Meng Shao, Jian Chen, Guang-sheng Luo, Jian-hong Xu

**Affiliations:** 1 The State Key Lab of Chemical Engineering, Department of Chemical Engineering, Tsinghua University, Beijing, 100084 (China)

## Abstract

Here in this article, we classify and conclude the four morphologies of three-phase emulsions. Remarkably, we achieve the reversible transformations between every shape. Through theoretical analysis, we choose four liquid systems to form these four morphologies. Then monodispersed droplets with these four morphologies are formed through a microfluidic device and captured in a petri-dish. By replacing their ambient solution of the captured emulsions, *in*-*situ* morphology transformations between each shape are achieved. The process is well recorded through photographs and videos and they are systematical and reversible. Finally, we use the droplets structure to form an on-off switch to start and shut off the evaporation of one volatile phase to achieve the process monitoring. This could be used to initiate and quench a reaction, which offers a novel idea to achieve the switchable and reversible reaction control in multiple-phase reactions.

Emulsification is a powerful method to disperse and mix multiple immiscible liquids. Multiple emulsions which are composed of multiple immiscible phases have the advantages of integrating different components and varied geometries in one system^1^. They could be used as templates to form multiple materials which could be applied in the biological analysis^2^, drug delivery^3^, microreactions^4^, and medicine^5^. Researchers have been working on adjusting the sizes, morphologies, and components of multiple emulsions. The sizes are usually controlled by the microfluidic technology. The microfluidics, raised from the 1990s, have been widely applied in synthesizing monodispersed and size-controllable emulsions^6^,^7^. Morphologies and components are the two vital factors that influence the applications of emulsions and they are strongly related. The morphologies of emulsions, in the thermodynamic state, are decided by the least interfacial energy theory which is related with the interfacial tensions between different components^8^,^9^. The morphologies and the components both have significant influence in the applications of emulsions. Core-shell emulsions have shells which could offer hinders and the cores which could resolve active substances thus the core-shell structure is one of the perfect morphologies to be utilized in the drug delivery field. Meanwhile, to apply in the drug delivery, the responsive components such as pNipam^3^ (one common thermo-responsive material) or chitosan^10^ (one common pH-responsive material) are needed. Janus emulsions, because of their natural asymmetric ability in the compositions and the shapes, are often used in the fields that need asymmetry in the shape and the materials. In these Janus materials, organic-inorganic composite^11^, nanoparticles^12^, and gas bubbles^13^ are often used to achieve specific functions. But the close relationship between morphologies and the components might restrict the application of specific components combination or restrict the components choice range for the specific morphology.

To reduce the restrictions, researchers have been working on the transformation between different morphologies. In the beginning, researchers changed the flow rates of different phases to control the morphology of Janus structure^14^ or the separate core size and shell size of the core-shell structure^15^. Then they combine the UV light and the kinetic process to obtain the equilibrium and nonequilibrium states of one system^16^. They also used the reaction to change the components to achieve the change of the interfacial tension between different phases to prepare reversible and dispersible polystyrene by adding N_2_ or CO_2_^4^,^17^. With calculations, researchers start to find that the Janus morphologies are just decided by the interfacial tension and flow rates ratio. They also used a facile and simple method to adjust their morphologies^18^,^19^. Researchers^20^ firstly used the surfactants to achieve tunable interfacial tensions to form dynamically reconfigurable double emulsions. By using the light-responsive surfactants, the emulsions transformed from core-shell to reverse core-shell structure. The dynamical transformation from Janus to core-shell evolution is also achieved in flowing state^21^. Small particles, which could disperse at the interface of different phases, also have been used to achieve the reconfiguration of bubble core-shell structure to Janus structure^12^. From all these results, we found that the separate transformation between different Janus structures, core-shell to reverse core-shell structures, Janus to core-shell structures, or core-shell to Janus structures have been achieved by different research groups.

Also, for the Janus structures, researchers renamed them with different names to show Janus specific morphology rather than concluding their functions according to their applications. Dumbbell Janus structure^22^,^23^, which is also called snow-man Janus^24^ or mushroom-type Janus^25^, is often used to describe the Janus emulsion that is a nonspherical shape with two connecting parts. The dumbbell Janus emulsions are used as templates to form catalysts, surfactants replacements, multiple functional materials^26^, inorganic-organic polymers^11^ and other nonspherical microparticles^13^. Another typical Janus structure, which is named as perfect Janus, is a spherical shape with asymmetric components in different directions^27^. These perfect Janus droplets are used as optical materials, such as dual-driven particles for electronic paper^28^ and the flexible beads with fluorescent responsivity^26^. From previous descriptions, most researchers focus only one morphology. The connection and transformation between the dumbbell structure and the perfect structure has not been studied. The study between them could bring an uniform understanding in distinguishing the emulsion morphologies.

Here in this article, we classify and conclude all the four morphologies of three-phase emulsions. Through analysis, we choose four liquid systems to form these four morphologies. The droplets are formed through a microfluidic device and captured in the petri-dish. By replacing their ambient solution of the captured droplets, *in*-*situ* morphology transformations between every shape are achieved. The process is well recorded through photographs and videos, and they are systematical and reversible. Finally, we use the droplets structure to form an on-off switch to start and shut off the evaporation of one volatile phase to achieve the process monitoring. This could be used to initiate and quench a reaction, which offers a novel idea to achieve the switchable and reversible reaction control in multiple-phase reactions.

## Results and Discussions

**Figure 1:**
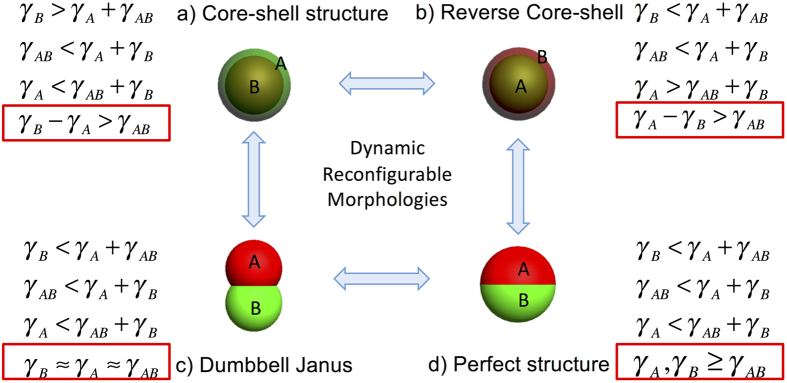
The transformation process between each structure and their interfacial relationships. The structures include: (**a**) Core-shell structure. (**b**) Reverse core-shell structure. (**c**) Dumbbell Janus structure.(**d**) Perfect structure.

According to the lowest interfacial energy and the geometry analysis, there are four morphologies for double emulsions. They are: (a) Core-shell structure (b) Reverse core-shell structure (c) Dumbbell Janus structure (d) Perfect Janus structure. They are classified by the interfacial tension relationships. The interfacial tension relationships between liquids, the corresponding morphology diagrams, and their transformation processes are shown in Fig. 1. Here in this article, *γ*_*A*_ represents the interfacial tension between A phase and the continuous phase (ambient phase), *γ*_*B*_ represents the interfacial tension between B phase and the continuous phase (ambient phase), and *γ*_*AB*_ represents the interfacial tension between A phase and B phase. It is worth noting that the equations are raised in the 1970s^8^ and here we deduct and simplify them to classify and conclude the four morphologies because the ordered and specific classification contributes to the emulsion applications. After simplification, each morphology can be decided by the equation with a red rectangle in Fig. 1. The derivation processes of equations are shown in the supporting information (Fig. S1).

Here in this article, we synthesize all the above morphologies from our microfluidic device and research on their reconfigurable transformations. We use the double-core capillary microfluidic device to form the droplets and then collect them into a cap which is used to capture the emulsions in the petri-dish. Then we replace the ambient solution of the droplets in the petri-dish through Polytetrafluoroethylene (PTFE) pipe by pumping in new ambient solution and extracting out original ambient solution. After replacing the ambient solution, the emulsions *in*-*situ* transform to another shape. To observe the process, microscope connecting to the high-speed camera is used to record videos. The operation process diagram is shown in the supporting material. The details of the microfluidic device are shown in the **Method** section.

To select the liquid systems to achieve the four morphologies and their reconfiguration process, the interfacial tensions between many liquids are measured. The interfacial tension between liquids is measured through the pendent drop method with a dynamic interfacial tension measurement instrument (OCAH200, DataPhysics Instrument GmbH, Germany). Because there are surfactants in the liquids, the measurement is dynamic through the process until the dynamic interfacial tension keeps stable. Then we calculate the average value of the stable parts which conclude dozens of measurement results of the interfacial tensions. We use the average value as the interfacial tension between liquids. The instrumental error is ±0.1 mN/m. After analysis, four liquid systems are selected (See Table S1). The selection process of the liquid systems used in the experiments is shown in the supporting material (Fig. S2). Every transformation process has its liquid system. The system includes one A phase, one B phase, and two continuous phases. It is the change of the continuous phase that leads the morphology transformation.

**Figure 2:**
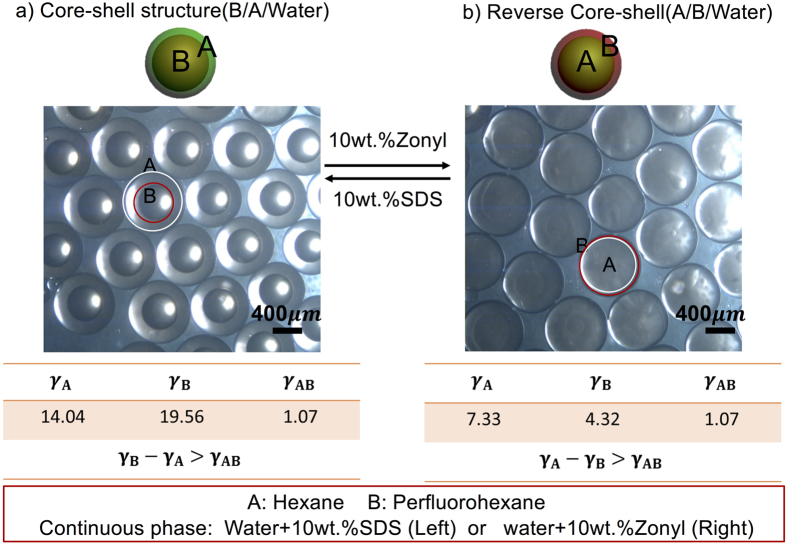
The transformation process between core-shell structure and the reverse core-shell structure. The systems are hexane (**a**), perfluorohexane (**b**), and the continuous phase which is deionized water with 10 wt.% SDS (Core-shell structure) or deionized water with 10 wt.% Zonyl (Reverse Core-shell structure). The interfacial tensions of the liquids are also shown in the image. The scale bar represents 400 μm.

The first transformation process is between core-shell structure and the reverse core-shell structure. The system is the same as the one in the ref. 20. To make the transformation process between four morphologies integrated, we do the transformation process using our device and experiment process. And the results achieved are shown in Fig. 2. The reconfigurable movies of the droplets are shown in supporting material (Movies S1 and S2). Here in the system, A is hexane; B is perfluorohexane and the continuous phase is deionized water with 10 wt.% SDS (Core-shell structure) or 10 wt.% Zonyl (Reverse Core-shell structure). When the continuous phase is deionized water with 10 wt.% SDS, the emulsion morphology is core-shell morphology with B inside because *γ*_*A*_ is smaller than *γ*_*B*_. After replacing the ambient solution to deionized water with 10 wt.% zonyl, *γ*_*A*_ and *γ*_*B*_ both decrease but *γ*_*B*_ decreases more so it is smaller than *γ*_*A*_. Thus, B phase extrudes to engulf A phase to form a reverse core-shell structure with A inside.

**Figure 3:**
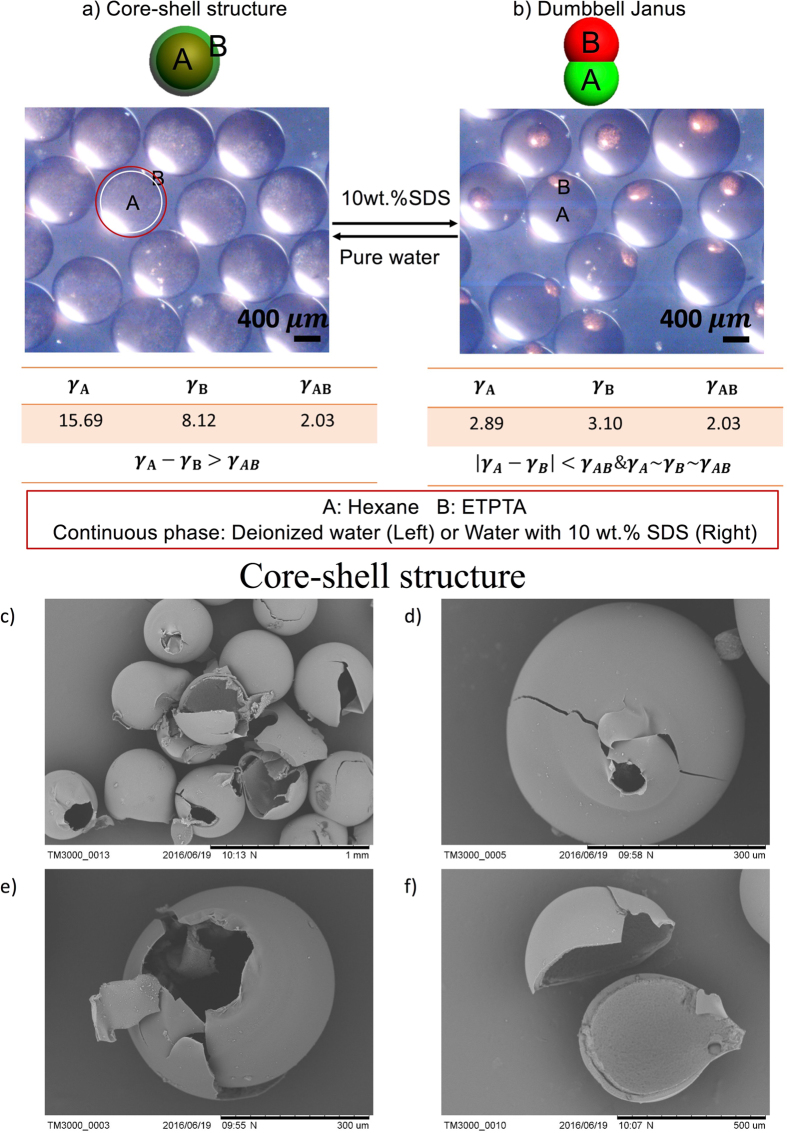
The transformation process between the core-shell structure and dumbbell Janus. The systems are hexane (A phase), ETPTA (B phase), and the continuous phase which is deionized water (Core-shell structure) or deionized water with 10 wt.% SDS (Dumbbell Janus structure). ETPTA is coloured red for distinction. The scale bar is 400 μm. (**c**–**f**) The SEM images of core-shell structures. ETPTA phase is the shell which is solidified while the hexane is not solidified thus the particles have cores inside. The hollows show their core-shell morphologies.

Figure 3 shows the transformation process between core-shell structure and dumbbell Janus structure. It is worth noting that we use the core-shell structure to represent core-shell and the reverse core-shell structure in the following text. Because the two structures are only different in the A and B phase definition and there is no need to distinguish the core-shell and the reverse core-shell structure when compared with Janus structure. The systems are hexane (A phase), Ethoxylated trimethylolpropane tri-acrylate (ETPTA, B phase), and the continuous phase which is deionized water (Core-shell structure) or deionized water with 10 wt.% SDS (Dumbbell Janus structure). ETPTA is a light-curable polymer and could be solidified under UV light. When the continuous phase is deionized water, the emulsion morphology is core-shell morphology with A inside because *γ*_*A*_ is larger than *γ*_*B*_. After replacing the ambient solution to deionized water with 10 wt.% SDS, *γ*_*A*_ and *γ*_*B*_ both decreases very fast to be similar with *γ*_*AB*_. Thus, the droplet morphology changes from core-shell structure to the dumbbell Janus structure. The core-shell structure in the microscopic image is not very clear because the shell is very thin. Here we solidify B phase (ETPTA) with UV light and check the structure by taking scanning electron microscope (SEM) images. After grinding the particles, we could find that the particles are hollow monodispersed particles (Fig. 3c–f). Thus it is clearly shown that the particles are core-shell structures. The reconfigurable videos of these two morphologies are shown in supporting material (Movies S3 and S4). The transformation process is very fast thus a microscope attached with a high-speed camera is used to record the dynamic transformation process (See Movie S5). And the video speed is one-twentieth of the real speed. From the video, we could find the morphology could be transformed in milliseconds.

**Figure 4:**
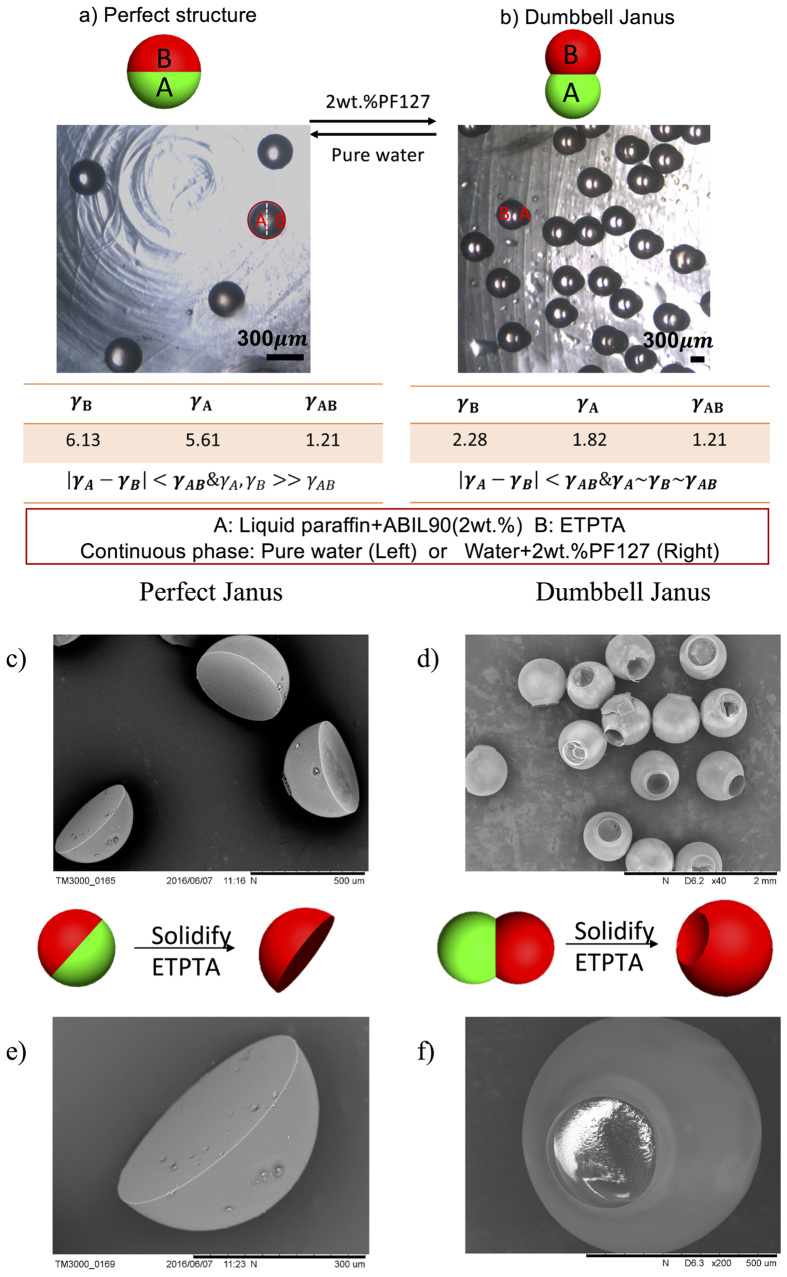
The transformation process between the perfect structure and the dumbbell Janus. The liquids are liquid paraffin with 2 wt.% ABIL-90 (A phase), ETPTA (B phase), and the continuous phases which are deionized water (Perfect Janus) and deionized water with 2 wt.% PF127 (Dumbbell Janus). ETPTA is coloured red for distinction. The scale bar is 300 μm. (**c**–**f**) Show the SEM pictures of the perfect Janus and Dumbbell Janus by solidifying ETPTA phase. (**c**,**e**) The SEM pictures of Perfect Janus when solidifying ETPTA. They form semi-sphere structure. (**d**,**f**) The SEM pictures of Dumbbell Janus when solidifying ETPTA. They form spheres with holes in one side because the two phases only connect a small part of each other.

Then we study on the transformation process between perfect Janus and dumbbell Janus structure. They are composed of liquid paraffin with 2 wt.% ABIL-90 (A phase), ETPTA (B phase), and the continuous phases which are deionized water (Perfect Janus) and deionized water with 2 wt.% PF127 (Dumbbell Janus). When the continuous phase is pure water, the γ_A_ and γ_B_ are relatively bigger than γ_AB_ to form a perfect Janus which is shown in Fig. 4a. After adding surfactants (2 wt.% PF127) into water, the γ_A_ and γ_B_ decrease to be relatively the same as γ_AB_ to form a dumbbell Janus, as shown in Fig. 4b. Their movies are shown in S6–S11. Also, we solidify the ETPTA phase to see their SEM images to confirm their morphologies. Figure 4(c,e) show the perfect Janus SEM images and Fig. 4(d,f) show the dumbbell Janus SEM images. The ETPTA forms semi-sphere particles when forming a perfect Janus morphology. When forming the dumbbell Janus morphology, the ETPTA forms a non-spherical shape with a hole in one side because the two phases only connect a small part of each other.

**Figure 5:**
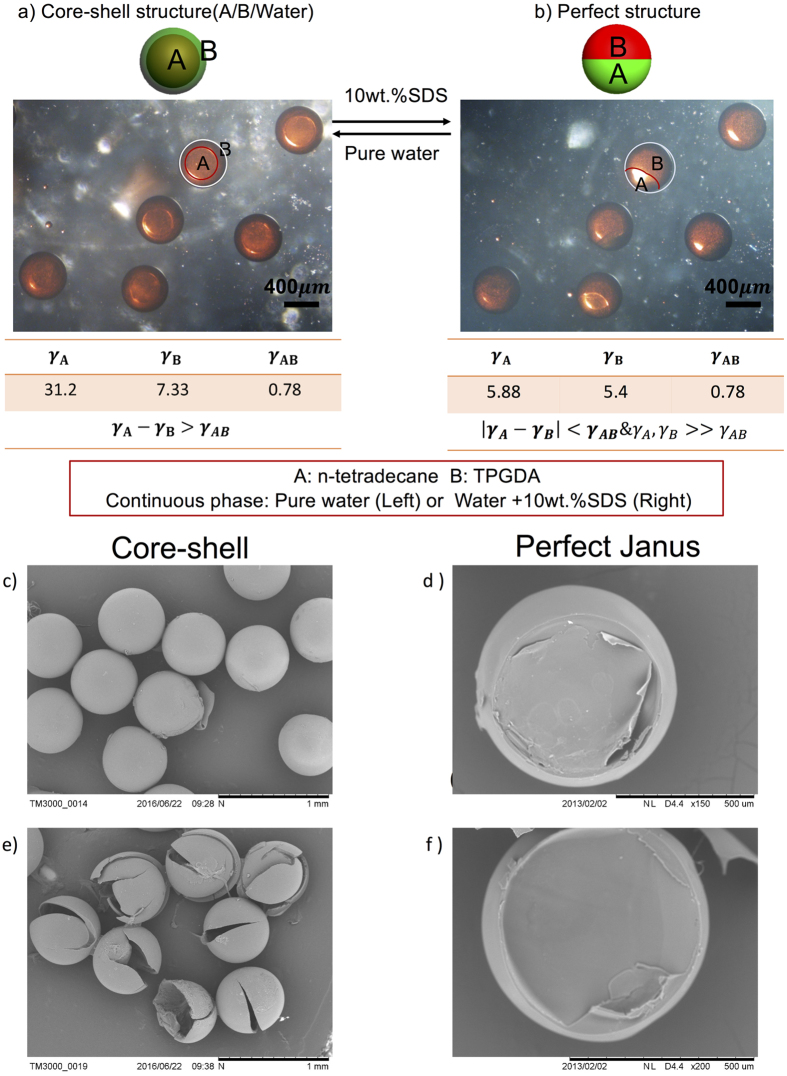
The transformation process between core-shell structure and perfect Janus structure. (**a**) Core-shell structure with A phase inside. (**b**) The perfect Janus structure. The liquids are n-tetradecane (A phase), TPGDA (B phase, light-curable polymers), and the continuous phase which is deionized water (Core-shell structure) or deionized water with 10 wt.% SDS (Perfect Janus). TPGDA is coloured red for distinction. The scale bar is 400 μm. (**c**,**e**) Show the SEM pictures of the core-shell structure. (**c**) The integral spheres observing from outside. (**e**) The broken particles show the holes inside the shells. (**d**,**f**) show the SEM pictures of the perfect Janus structure. They are half sphere particles when only solidifying TPGDA phase of the Perfect Janus.

The last transformation process is between core-shell structure and perfect Janus structure which is shown in Fig. 5. The liquids are n-tetradecane (A phase), tri-propylene glycol diacrylate (TPGDA, the B phase, light-curable polymers), and the continuous phase which is deionized water (Core-shell structure) or deionized water with 10 wt.% SDS (Perfect Janus). TPGDA is a light-curable polymer and could be solidified under UV light. When without surfactant, the interfacial tension between B and water is much smaller than that between A and water, thus B engulfs A to form a core-shell structure. After adding SDS, the γ_A_ and γ_B_ both decrease to meet the interfacial tension relationship of forming the Janus structure. And because γ_AB_ is smaller than γ_A_ and γ_B_, they form a perfect Janus structure. The reconfigurable movies of the droplets are shown in the supporting material (Movies S12 and S13). To confirm their structures, we solidify TPGDA to observe their SEM pictures. When forming a core-shell structure, TPGDA is the shell and when forming perfect Janus, TGPDA consists of one-half of the sphere. Figure 5 include the SEM images of these two structures. From Fig. 5(c,e), we observe that they are forming monodispersed spheres. After grinding the microparticles, we found that they are hollow particles with holes inside. That is because A phase (n-tetradecane) is not solidified. Figure 5(d,f) show the particles are half-spheres because we only solidified one-half of the two phases.

From the above results, we have achieved the reversible transformation between four typical structures of three-phase emulsion systems. Thus we systematically propose and synthesize the four different morphologies of three-phase emulsion systems. Remarkably, we achieve their *in*-*situ* transformations by simply replacing their ambient solutions. The emulsion systems in the article are in the equilibrium state that ensures the controllable and predictable transformation between different morphologies. This transformation could be widely used in fields for the diverse particle formation or the fields that need the transformation of the structures.

**Figure 6:**
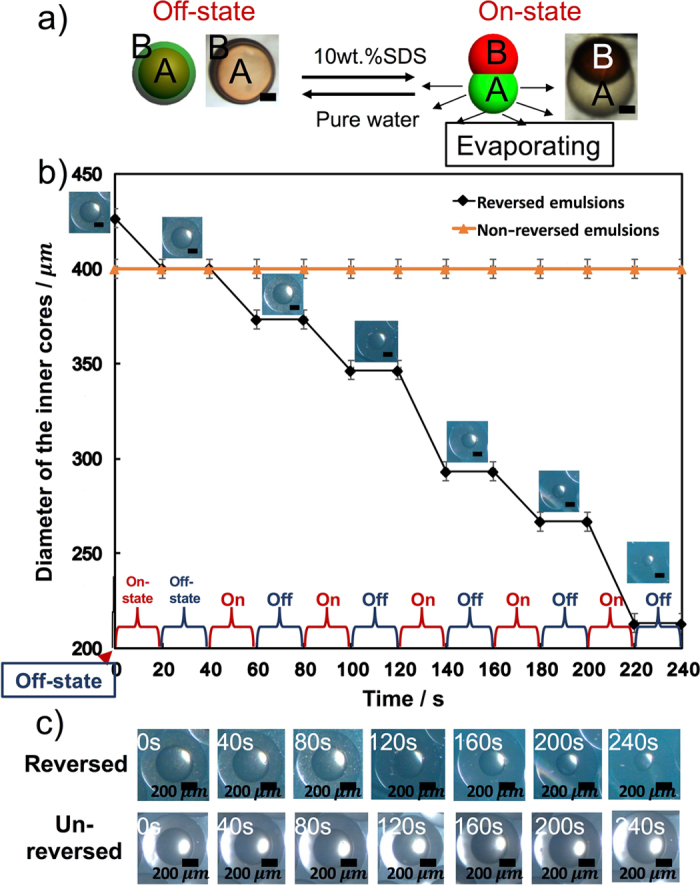
The on-off switch model to control the evaporation of the core. (**a**) The on-off states of the switch. The core is hexane and the shell is ETPTA. When the droplet is core-shell structure, the hexane stops evaporation (off-state) and when the droplet is Dumbbell Janus, hexane contacts to the environment to be in the on-state. (**b**) The alternate emulsion states changing with time. The emulsion states are the off-state (microscopic image is taken), the transition process to on-state, the on-state (20 seconds), the transition process to off-state, and the off-state (20 seconds, a microscopic image is taken), sequentialy. The process is repeated for six times. (**c**) The comparison between reversed droplets and un-reversed droplets in the diameters of inner cores with time and their microscopic images. The scale bar represents 200 μm.

For instance, we utilize the reversible transformations to control the on-off state of the evaporation process. Figure 6 depicts the process of multiple transformations between the core-shell structure and the dumbbell Janus structure. The liquid phases are hexane, ETPTA, and deionized water with or without surfactants (SDS). At the beginning, the liquids consisting of hexane, ETPTA and deionized water form emulsions that are the core-shell structures with hexane inside. Hexane is easy to evaporate and ETPTA is nonvolatile. When the hexane is engulfed by ETPTA, the evaporation rate is lowest (off-state). When the emulsion transforms to dumbbell Janus structure, the haxane speed up its evaporation to be in an on-state. The on-state and the off-state morphologies and the transformation process is shown in Fig. 6a. Here we prepare two petri-dishes with the same core-shell structure emulsions. One is used to transform their morphologies by replacing its ambient solutions and getting reverse after every twenty seconds. Another one is replaced with its original ambient atmosphere to keep the droplets structure unchanged. For the reversed emulsion, the start state is the off-state (core-shell structure). Then it transforms to the on-state (dumbbell structure). The transformation process is very fast thus we could ignore the influence of the transformation process when recording the core sizes with time. After keeping in the on-state for 20 seconds, the emulsion is transformed to the off-state to take microscopic image which is used to measure the core size. Then the emulsion keeps the off-state for 20 seconds and transforms to the on-state. The process is repeated for six times to measure the change of the core size. The alternate states and the core sizes changing with time are shown in Fig. 6b. To ensure the accuracy of the inner core sizes, we calculate them in the off-state (the core-shell morphology). We could observe that as the transformation occurs every time, the core is becoming smaller and smaller as the hexane evaporates. Figure 6c shows the comparison between reversed droplets and non-reversed droplets in the diameters of inner cores with time and their microscopic images. We could find the sizes of inner cores of reversible droplets decrease after every reverse transformation while the sizes of the non-reversed droplets keep almost the same. This is how to use the on-off switch to control the evaporation. When the double emulsion is core-shell structure, the switch is in the off-state which means the evaporation is quenched. After replacing the ambient solutions, the morphology transforms to the Dumbbell Janus structure which is in the on-state because the hexane connects to the environment to speed up evaporation. This method responses very fast and does not introduce unrelated components. Also it is reversible and controllable. The on-off switch to control the evaporation process gives us the example to show its potential application to initiate and quench reactions.

In conclusion, we raise the idea of the reconfiguration between four morphologies of three-phase emulsions. The four morphologies are: (a) Core-shell structure (b) Reverse core-shell structure (c) Dummbell Janus (d) Perfect Janus. The morphologies are in the equilibrium states which are decided by the interfacial tensions between liquids. Remarkably, the reconfigurable transformation processes between each morphology are achieved and elaborately recorded. Also, we use the reconfigurable morphology transformation to control the evaporation process to show its potential application in working as an on-off switch. The switch could well control the evaporation process with fast responsivity. This article offers in-depth understanding of the dynamic and reversible morphologies transition of three-phase emulsions. Their controllable reconfiguration offers the solid support on the formation of smart materials with diverse structures and applications.

## Methods

### Materials

The oil phases we used include Hexane, Perfluorohexane, Ethoxylated trimethylolpropane tri-acrylate (ETPTA, purchased from Aladdin, Co. Ltd.), liquid paraffin, and tri-propylene glycol diacrylate (TPGDA). Deionized water is used to work as the water phase. Surfactants are used to adjust the interfacial tensions between liquids. ABIL90 (Purchased from Evonik) and Sorbitan oleate 80 are used to add into the oil phase while the sodium dodecyl sulfate and Pluronic F-127 (purchased from Aladdin Co. Ltd.) are used to add into the water phase. They are chemically pure. ETPTA and TPGDA are light-curable polymers. Here we use 2-Hydroxy-2-methylpropiophenone (HMPF, purchased from Sigma. Co. Ltd.) to work as photo initiator to initiate solidification of ETPTA and TPGDA with UV light. We use the microscopic machine to take microscopic images and record videos of the transformation process. If it might be difficult to ensure the morphologies through the microscopic images, we solidify one phase of the system to check their Scanning Electronic Microscopy (SEM) images.

### Devices

We use the double-core capillary microfluidic device to form the droplets and then collect them into the petri-dish. The microfluidic channel is composed of polymethyl methacrylate (PMMA) with the size of 25 mm (length) × 25 mm (width) × 5 mm (height). The tip of the double-core capillary is tipped with 50 μm of each core. And the outer size of the double-core capillary is 1.5 mm while the inner size of the PMMA is also 1.5 mm to ensure the coaxiality of the device. The joint of the tubes, capillaries and chips are sealed by glues. Other devices are micropipette puller (P-97, SUTTER Co. Ltd., USA) for pulling the capillary into a tip, micro-syringe pumps (LP01-1B, Longer Precision Pump Co. Ltd.) for pumping the liquids, the optical microscope (BX61, Olympus Co. Ltd.) for taking microphotographs and movies, and a dynamic interfacial tension measurement instrument (OCAH200, DataPhysics Instrument GmbH, Germany) for measuring the interfacial tension between the liquids. The interfacial tension between liquids is measured through the pendent drop method. Because there are surfactants in the liquids, the measurement is dynamic through the process until the dynamic interfacial tension keeps stable. Then we calculate the average value of the stable range as the interfacial tension between liquids. We take the Scanning Electronic Microscopy (SEM) images through SEM machine (TM3000, Hitachi Co. Ltd.).

## Additional Information

**How to cite this article**: Ge, X.- *et al*. Four reversible and reconfigurable structures for three-phase emulsions: extended morphologies and applications. *Sci. Rep.* **7**, 42738; doi: 10.1038/srep42738 (2017).

**Publisher's note:** Springer Nature remains neutral with regard to jurisdictional claims in published maps and institutional affiliations.

## Supplementary Material

Supporting Information

Supplementary video S1

Supplementary video S2

Supplementary video S3

Supplementary video S4

Supplementary video S5

Supplementary video S6

Supplementary video S7

Supplementary video S8

Supplementary video S9

Supplementary video S10

Supplementary video S11

Supplementary video S12

Supplementary video S13
