# Supplementary material for: Four reversible and reconfigurable structures for three-phase emulsions: extended morphologies and applications
Source: Sci Rep. 2017 Feb 15;7:42738. doi: 10.1038/srep42738 (PMC5309921; doi:10.1038/srep42738)
Supplement: Supporting Information [file srep42738-s1.pdf]

Four reversible and reconfigurable structures for three-phase emulsions: extended morphologies and applications

Xue-hui Ge, Yu-hao Geng, Qiao-chu Zhang, Meng Shao, Jian Chen, Guang-sheng Luo and Jian-hong Xu *

Supporting information package

The package uploaded includes 1 word file and 13 video files.

They are:

One word file:

1. *Supporting information.docx.*

It describes the equations and analysis process.

Thirteen Videos files:

S1-Core-shell structure to the reverse core-shell structure

This file recorded the dynamic motions of the in-situ transformation from the core-shell structure to the reverse core-shell structure.

S2-Reverse core-shell to core-shell structure

This file recorded the dynamic motions of the in-situ transformation from the reverse core-shell structure to the core-shell structure.This is the reverse process of S1

S3-Core-shell structure to dumbbell Janus

This file recorded the dynamic motions of the in-situ transformation from Core-shell structure to dumbbell Janus structure.

S4-Dumbbell Janus to core-shell structure

This file recorded the dynamic motions of the in-situ transformation from dumbbell Janus structure to Core-shell structure. This is the reverse process of S3

S5-Core-shell to dumbbell Janus in dynamic tube

This file recorded the dynamic motions of the in-situ transformation from dumbbell Janus structure to Core-shell structure in a tube with flowing continuous liquids.

S6-Dumbbel Janus to perfect Janus

This file recorded the dynamic motions of the in-situ transformation from Dumbbell Janus to perfect Janus.

S7-Perfect Janus formation

This file recorded the droplets formation process of perfect Janus structures.

S8-Dumbbell Janus formation

This file recorded the droplets formation process of Dumbbell Janus structures.

S9-Dumbbell Janus droplets

This file recorded the droplets of Dumbbell Janus structures.

S10-Perfect Janus droplets

This file recorded the droplets of Perfect Janus structures.

S11-Perfect Janus to dumbbell Janus

This file recorded the dynamic motions of the in-situ transformation from Dumbbell Janus to perfect Janus in the tube with flowing continuous phase.

S12-Core-shell to perfect Janus

This file recorded the dynamic motions of the in-situ transformation from Core-shell structure to perfect Janus structure.

S13-Perfect Janus to core-shell structure

This file recorded the dynamic motions of the in-situ transformation from Perfect Janus to core-shell structure. This is the reverse process of S12

Supplementary information

1. Equation deviation

In 1967,Torza[1] discovered the relationship between spreading coefficients and morphologies.With time proceeding, the understanding of the morphologies becomes more specific because of the intense needs of the particle applications. The morphologies of the three phase system include core-shell structure,Janus structure and separating structure.

When applied in the researches and industries, the separating structure is regarded as two separating droplets and not regarded as multiple emulsions.For core-shell structures,the core and the shell materials are the main considerations for applications. When using this structure, which is the core and which is the shell is initial in the choosing of ambient atmosphere and the actives' hydrophobicity or hydrophilicity.Thus we separate the core-shell structure into core-shell structure and reverse core-shell structure to distinguish the components of the core/shell.

As for the Janus structures,the integral structure(sphere or non-sphere) and the connecting condition of two phases are most considered.In my previous work,we have achieved the methodology of designing Janus structure with simulation in the interfacial tensions between three phases and the flow ratio.But in the most researches and industry applications, they care first about the integral structure of the Janus.Jan Guzowski[2] first describe the requirement for forming the perfect-state Janus by analyzing the interfacial tension relationship between three liquid phases. After that,researchers have named the spherical Janus morphology as perfect Janus. On the contrary, dumbbell Janus/snow Janus which describes one non-sphere structure composed of two lobes. Here in this paper,we unify these names as perfect Janus and dumbbell Janus and conclude them through more fundamental methods:the interfacial tension relationship.Then we used this relationship to choose and adjust liquid phases to form these two morphology droplets and proceed their transformation.

They are decided by the contact angle in the Newman Triangle model. α,. β and . δ are determined by interfacial tensions γ_A, γ_B andγ_{AB}, according to the following equation:

$$\alpha = acos\left[0.5(\gamma_A^2 + \gamma_{AB}^2 - \gamma_B^2) / (\gamma_A \times \gamma_{AB})\right] \quad (1)$$

$$\beta = acos\left[0.5(\gamma_A^2 + \gamma_B^2 - \gamma_{AB}^2) / (\gamma_A \times \gamma_B)\right] \quad (2)$$

$$\delta = acos\left[0.5(\gamma_B^2 + \gamma_{AB}^2 - \gamma_A^2) / (\gamma_B \times \gamma_{AB})\right] \quad (3)$$

When $\gamma_A \sim \gamma_B \sim \gamma_{AB}$,we could calculate the angles roughly to be about 60° which would be a typical dumbell Janus.[2] When $\gamma_A, \gamma_B \gg \gamma_{AB}$, β would be close to zero which is a typical perfect Janus.It has a strict restriction on forming perfect Janus.Only those which meet the restriction of Janus structure and $\gamma_A, \gamma_B \gg \gamma_{AB}$it would form perfect Janus.When γ_A, γ_B are higher than γ_{AB},the Janus is more perfect.[2] The three categories according to Torza and the specific classification on the Janus as well as the contacting angles relationship is shown in Figure S1.

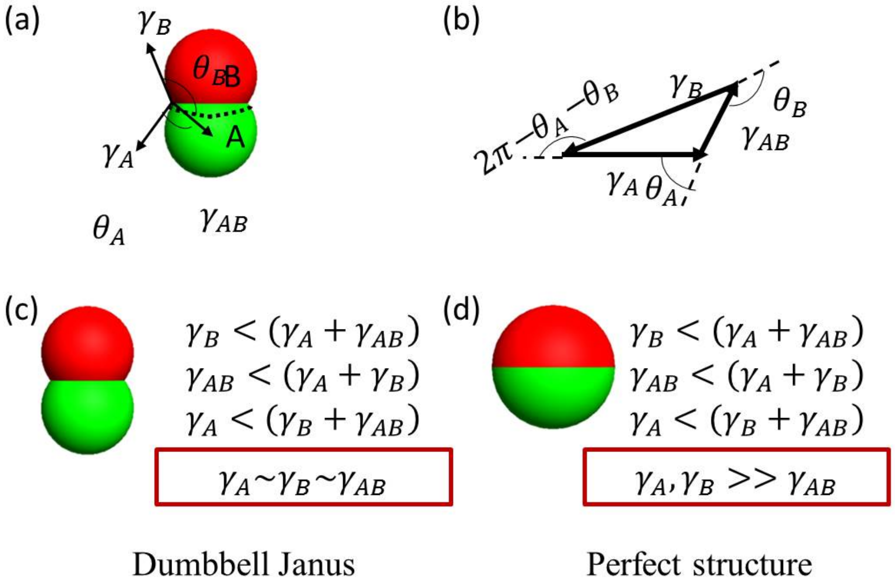

Figure S1. The droplets morphologies with restrictions in their interfacial tensions. (a) Janus contact angles with interfacial tensions. γ_A represents the interfacial tension between A phase and the continuous phase. γ_B represents the interfacial tension between B phase and the continuous phase. γ_{AB}, represents the interfacial tension between A phase and B phase. (b) Newman Triangle about the contacting angle and the interfacial tensions. (c) Dumbbell Janus. (d) Perfect Janus.

As the core-shell and reverse core-shell structure, the distinction is the value of γ_B and γ_A. When

γ_B is larger, B phase is inside and when γ_A is larger, A phase is larger.

According to the core-shell interfacial tension relationship, the equations are shown as:

$$\gamma_B > \gamma_A + \gamma_{AB} \quad (4)$$

$$\gamma_{AB} < \gamma_A + \gamma_B \quad (5)$$

$$\gamma_A < \gamma_{AB} + \gamma_B \quad (6)$$

Equation (4) could be adjusted to:

$$\gamma_B - \gamma_A > \gamma_{AB} \quad (7)$$

Thus, for simplification, we can conclude that:

when $\gamma_B - \gamma_A > \gamma_{AB}$, it means γ_B is larger and B phase is inside to form a core-shell structure;

When $\gamma_A - \gamma_B > \gamma_{AB}$, it means γ_A is larger and A phase is inside to form a reverse core-shell structure;

2.Selection of the liquid systems

To achieve the transformation of these morphologies, we have to apply for our working systems and operation orders strictly according to the theory and the logic analysis. Here we take the transformation from the perfect Janus to dumbbell Janus as an example. From the previous analysis between the morphology and the interfacial tension, we know that other than meeting the needs to form Janus droplets, to form a Perfect Janus, the working liquids should meet $\gamma_A, \gamma_B >> \gamma_{AB}$ while forming a dumbbell Janus, the working liquids should meet $\gamma_A \sim \gamma_B \sim \gamma_{AB}$. Thus, to change from one to another, we have four possible ways, which is shown in Figure S2(a), that is:

1) Decrease the big γ_A, γ_B to small γ_A, γ_B which approach to small γ_{AB} to achieve from Perfect Janus to Dumbbell Janus;
2) Decrease the big γ_{AB} which has been relatively the same to big γ_A, γ_B to approach to small γ_{AB} to achieve from Dumbbell Janus to perfect Janus;
3) Increase the $small\ \gamma_A, \gamma_B$ which has been relatively the same to the small γ_{AB} to big γ_A, γ_B to achieve from Dumbbell Janus to Perfect Janus;
4) Increase the small γ_{AB} to approach to big γ_{AB} which is relatively to big $\gamma_A, \gamma_B$to achieve from Perfect Janus to Dumbbell Janus.

When applying into real working systems, we usually use two immiscible oil phases and one water phase to work as system liquids with surfactants to change the interfacial tension between water and oil. For these systems, four combinations can be listed which shown in Figure 2 (b).

a) A and B are oil phases and the continuous phase is water. Then we use surfactants in water to decrease the γ_A, γ_B;
b) is oil and B is water while continuous phase is another oil. Then we add a surfactant into B phase to decrease the γ_{AB};
c) A is oil and B is water while continuous phase is another oil. Then we add a surfactant into continuous oil to change γ_B or γ_{AB};
d) A and B are both oil phase while the continuous phase is water. Then we add a surfactant into oil phase (A or B) to change the γ_A or γ_B.

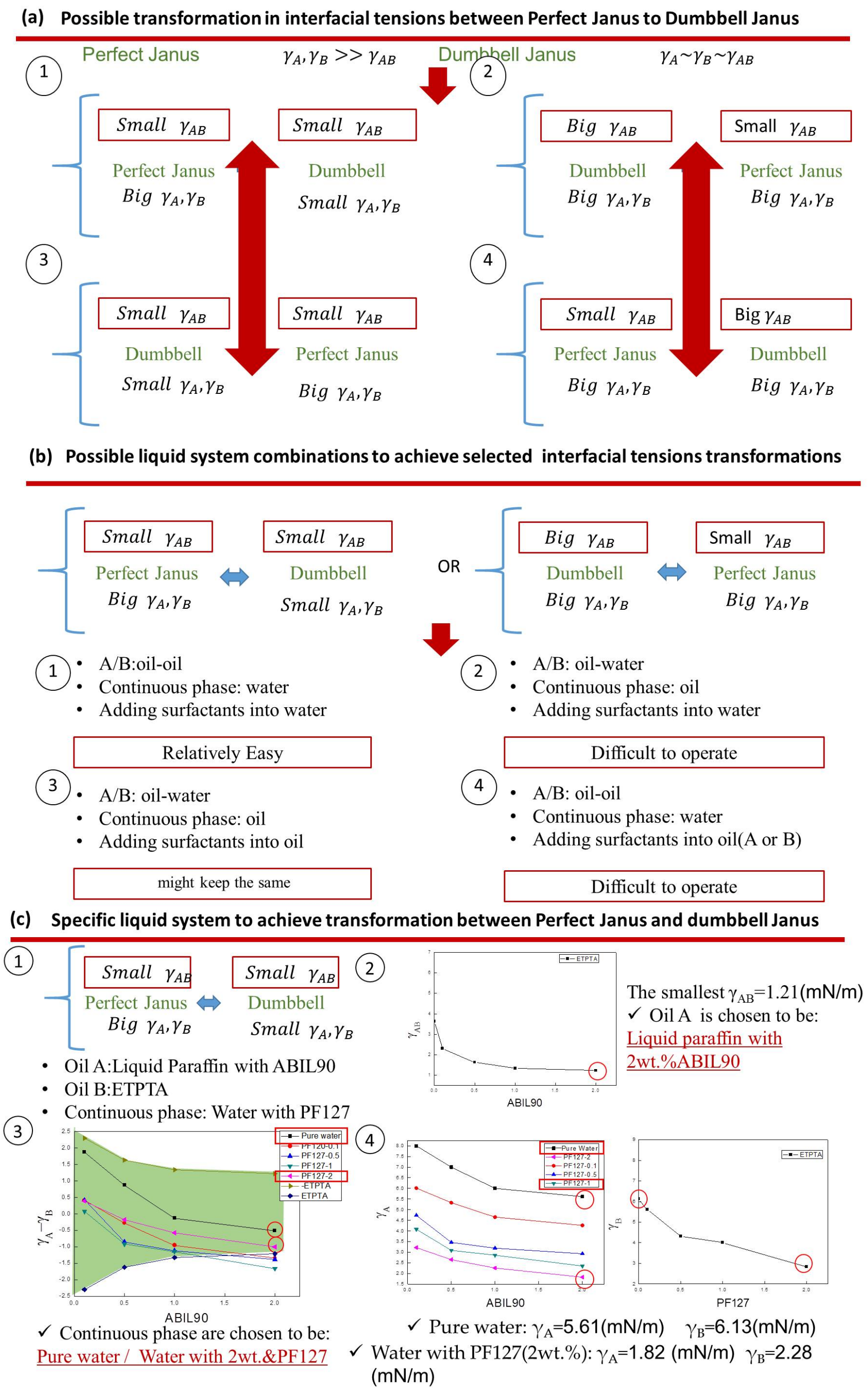

Figure S2. (a)The four possible ways to transform between Perfect Janus and Dumbbell Janus structures. 1) Keeping small γ_{AB} and changing big γ_A, γ_B to small γ_A, γ_B; 2) Keeping big γ_A, γ_B and changing bigγ_{AB} to small γ_{AB}; 3) Keeping small γ_{AB} and changing small γ_A, γ_B to big γ_A, γ_B; 4) Keeping big γ_A, γ_B and changing small γ_{AB} to big γ_{AB}. For these four possible ways, 1) and 3) are the transformation between big γ_A, γ_B to small γ_A, γ_B and keeping the small γ_{AB} while 1) and 4) are the transformation between big γ_{AB} to small γ_{AB} and keeping the big γ_A, γ_B. (b) The analysis tree of the working system transformed from Perfect Janus and Dumbbell Janus. 1) A and B are oil phases while the continuous phase is water phase with or without surfactant to achieve the transformation between big γ_A, γ_B to small γ_A, γ_B. The continuous phase is relatively simpler to replace, thus this method is relatively easy.2) A and the continuous phase are oil while B is water with

or without surfactant to adjust the value of γ_{AB} .The problem might be that it is difficult to add or remove surfactants in the dispersed oil phase after forming droplets to achieve in-situ droplets reconfiguration. 3) A and the continuous phase are oil while B is water with or without surfactant to adjust the value of γ_{AB} or γ_{B} .The problem is that γ_{A} might keep the same that fail to achieve the transformation. 4) A and B are oil phases while the continuous phase is water. Here the surfactant is added into one oil phase(A or B) to adjust γ_{A} or γ_{B}.The problem might also be difficult to operate and fail to achieve the transformation. Thus through the enumeration method, we could conclude that the optimum system combination is two dispersed oil phase and the continuous water phase with or without surfactant.(c) 1) The transformation of the interfacial tensions and the possible liquid systems. 2) The interfacial tension between ETPTA and liquid paraffin with the increasing concentration of ABIL90 in the liquid paraffin. The target of this procedure is to choose oil A and oil B. 3) The Interfacial tensions relationship between different continuous phases with the increasing concentration of ABIL90 in the liquid paraffin. The target of this procedure is to choose two continuous phases. 4) The check on the interfacial tensions of two chosen systems.

While in real operation, it is relatively difficult to add a surfactant into dispersed phases which have been sheared to be one part of droplets. Thus we could ignore the methods above of adding surfactants into dispersed phases. As for the condition of adding surfactants into the continuous oil phase, it has the problem that it just change the interfacial tension between water and oil rather than that between two oil phases. That is, γ_A might keep unchanged while γ_B is decreasing largely.

To make all the logic analysis a conclusion, we found that the system composed of two oil phases working as A and B and water working as the continuous phase fits the reality best. When operating, we only need to replace the pure water with water solution adding surfactants or do the reverse.

With the analysis in the system combination, it has been listed to choose the oil/oil/water with(without) surfactants to achieve the transformation between Perfect Janus and Dumbbell Janus structures. Here we choose ETPTA and Liquid paraffin as oil phases and water as continuous phase. ABIL 90 is an oil-soluble surfactant and PF-127 is water-soluble surfactant to adjust the interfacial tensions. In this system, the γ_{AB} needs to be small (the smaller, the better), and the γ_A and γ_B need to be changed from big to small as shown in Figure 2(c1). Firstly we measured the interfacial tension between ETPTA and liquid paraffin (γ_{AB})) with the increasing concentration of ABIL90. From Figure 2(c2), we could find the γ_{AB} is decreasing with the increased concentration at the beginning and approaching to be stable when the concentration is more than 1 wt. %. Here we choose the concentration of ABIL 90 to be 2 wt. % when γ_{AB} equals 1. 21 mN/m. Then we measure the γ_A and γ_B in the different ABIL 90 and different PF127 concentration and calculate $\gamma_A - \gamma_B$ to draw its relationship with ABIL90 concentration. In Figure 2(c3). The green area represents the dots (systems) that could form Janus morphologies because they meet the needs of $-\gamma_{AB} < \gamma_A - \gamma_B < \gamma_{AB}$. In these dots, we found that when the ABIL 90's concentration is 2 wt. %, the continuous phase is pure water or water with 2 wt. % PF127 could form Janus structure. Then we turn to Figure 2(c4) to check their number. From the measurement , we found that when the continuous phase is pure water, γ_A=5.61 mN/m, γ_B=6.13 mN/m. They are relatively bigger than the γ_{AB}=1. 21 mN/m. Through simulation, the system might form perfect Janus. When the continuous phase is water with 2 wt. % PF127, γ_A=1. 82 mN/m , γ_B=2.

28 mN/m which are relatively the same with the γ_{AB}=1. 21 mN/m. The system might form dumbbell Janus. Thus we could try this system as one of our transformation systems.

To make a conclusion, the systems we used in the manuscript is shown in Table S1.

Table S1. The four liquid systems to achieve reconfigurable emulsions.

	A Phase	B Phase	Continuous Phase
**1. Core-shell structure to Reverse Core-shell structure**			
**Core-shell structure**	Hexane	Perfluorohexane	Deionized water with 10 wt. % SDS
**Reverse Core-shell structure**	Hexane	Perfluorohexane	Deionized water with 10 wt.% Zonyl
**2. Core-shell structure to Dumbbell Janus structure**			
**Core-shell structure**	Hexane	ETPTA	Deionized water
**Dumbbell Janus**	Hexane	ETPTA	Deionized water with 10 wt. % SDS
**3. Dumbbell Janus to Perfect Janus**			
**Dumbbell Janus**	Liquid paraffin with 2 wt.% ABIL-90	ETPTA	Deionized water with 2 wt.% PF127
**Perfect Janus**	Liquid paraffin with 2 wt.% ABIL-90	ETPTA	Deionized water
**4. Core-shell structure and Perfect Janus structure**			
**Core-shell structure**	n-tetradecane	TPGDA	Deionized water
**Perfect Janus structure**	n-tetradecane	TPGDA	Deionized water with 10 wt.% SDS

3.The experiment process diagram

We use the double-core capillary microfluidic device to form the droplets and then collect them into a cap which is used to capture the emulsions in the petri-dish. Then we replace the ambient solution of the droplets in the petri-dish through Polytetrafluoroethylene (PTFE) pipe by pumping new ambient solution and extracting original ambient solution. After replacing the ambient solution, the *in-situ* morphology transform to another shape. To observe the process, microscope connecting to the computer is used to record videos. The process is shown in **Figure S3.**

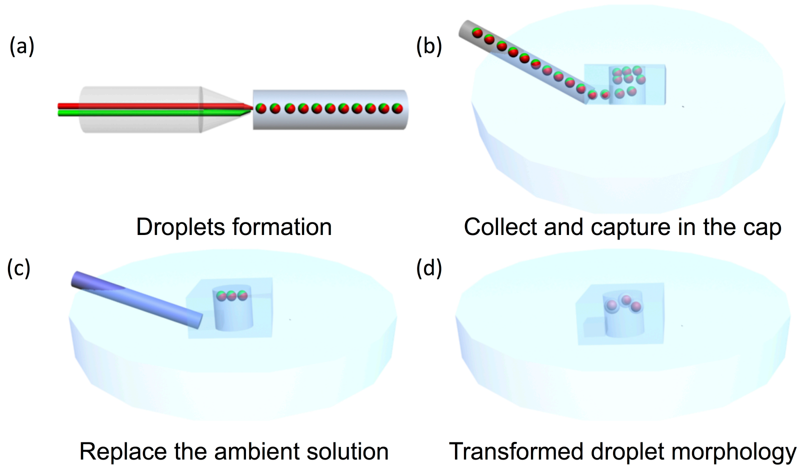

Figure S3.The droplets formation and the observation. Here we take the formation of Janus droplets and its transformation process to the core-shell droplets as an example. The two phases are coloured into green and red for distinction. (a)The Janus droplets are formed. (b) The Janus droplets are collected into a dish and trapped in a cap. (c) Replacing the ambient solution. (d) The droplets transform to core-shell droplets.

References

[1] S. Torza, S. G. Mason, *Science* 1969, *163*, 813-814.

[2] J. Guzowski, P. M. Korczyk, S. Jakiela, P. Garstecki, *Soft Matter* 2012, *8*, 7269.
